# An environmentally sustainable plasticizer toughened polylactide

**DOI:** 10.1039/c7ra13448g

**Published:** 2018-03-26

**Authors:** Hailan Kang, Yushi Li, Ming Gong, Yilin Guo, Zhuo Guo, Qinghong Fang, Xue Li

**Affiliations:** College of Materials Science and Engineering, Shenyang University of Chemical Technology Shenyang 110142 China fqh80@126.com; Department of Chemistry and Textile Engineering, Jiaxing University Nanhu College Jiaxing 314001 China lixue850704@163.com

## Abstract

Cardanol (CD), derived from renewable natural cashew nutshell liquid, has been used as a new plasticizer for polylactide (PLA), to create blends which retain the environmentally friendly features of PLA. The differential scanning calorimetry (DSC), dynamic mechanical thermal analysis (DMTA) and scanning electron microscopy (SEM) results all reveal that PLA and CD show good miscibility at low CD content. CD significantly decreased the glass transition temperature and enhanced the crystallization ability of PLA, demonstrating good plasticizing efficiency with PLA. At 10 wt% CD, ultimate elongation and impact toughness increased to 472% and 9.4 kJ m^−2^, respectively, which represented improvements of 31-fold and 2.6-fold over the corresponding measurements for neat PLA. The plasticization effect of CD was also demonstrated by the decreased melt complex viscosity and shear storage modulus at lower CD content for the blends when compared with neat PLA. Thus, the investigated CD presents an interesting candidate for a PLA plasticizer, meeting “double green” criteria. No cytotoxicity was found for the blends and hence they may be suitable for biomedical applications.

## Abstract 2

Cardanol, derived from renewable resources, exhibits good plasticizing efficiency for PLA, meeting “double green” criteria.

## Graphical abstract

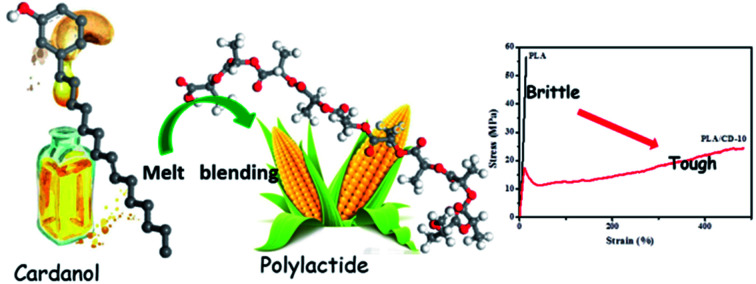

## Introduction

Polylactide (PLA), a most popular green plastic, provides a promising alternative to polymers derived from petroleum, and attracts much interest.^1–3^ Because of its renewability, biodegradability, biocompatibility and competitive physical properties, PLA has thus been applied in biomedical materials, packaging, and the automotive industry. However, despite all its suitable characteristics, PLA is limited by its inherent brittle nature and low crystallization ability.

Various approaches, including plasticization, copolymerization, and mixing with polymers or inorganic fillers, have been adopted to increase the toughness of PLA. PLA has been mixed with non-renewable and renewable polymers such as poly(ε-caprolactone),^4^ poly(ethylene oxide),^5^ polyurethane,^6,7^ polyamide,^8^ polyethylene,^9^ poly(butylene succinate),^10,11^ poly(butylene adipate-co-terephthalate),^12^ biobased polyesters,^13,14^ natural rubber^15,16^ and so on. Among the numerous toughening agents, plasticizers are preferentially used to reduce the brittleness of PLA. For example, several citrate esters, including triethyl citrate (TEC), tributyl citrate (TBC), acetyltriethyl citrate (ATEC), and acetyltributyl citrate (ATBC), have been used to modify PLA.^17^ All of the plasticized PLA blends exhibited a reduced glass transition temperature (*T*_g_) and an improved elongation at break. However, citrate esters with relatively low boiling points resulted in considerable weight loss during processing. Other ester-type plasticizers, such as glycerin triacetate (GTA) and bis(2-ethylhexyl) adipate (DOA), were effective in reducing the *T*_g_ of the blend but PLA plasticized with ∼25 wt% GTA^18^ or 20 wt% DOA^19^ exhibited phase separation. Oligomeric or polymeric plasticizers could reduce the migration and evaporation of the small molecule plasticizers and many studies have paid attention to the utilization of poly(ethylene glycol) (PEG) for PLA.^20,21^ The molecular weight as well as the additive amount of PEG influences the miscibility between PLA and PEG. The PLA/PEG blends with lower molecular weight PEG exhibited better miscibility, reducing the *T*_g_ to a greater extent. Modification of PLA has also been achieved *via* the addition of lactide monomers,^22^ soybean oil derivatives,^23,24^ castor oil derivatives,^25,26^ tartaric acid derivatives^27^ and so on. However, the challenge remains to find a new renewable plasticizer meeting the requirements of both a sustainable origin and biodegradable properties, to fulfill “double green” criteria.

**Fig. 1:**
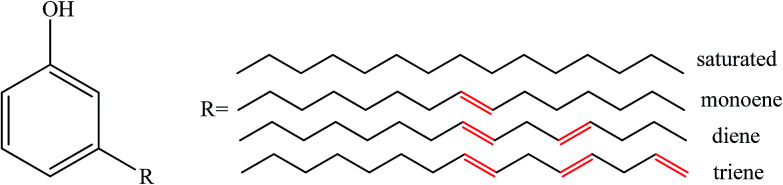
Structure of cardanol.

Cardanol (*m*-pentadecenyl phenol, CD) is a type of renewable agricultural by-product extracted from cashew nutshell liquid.^28^ CD has desirable features due to its chemical structure: (i) the benzene ring structure leads to high-temperature performance; (ii) the polar phenol hydroxyl group provides wetting and activity to the contact surface; and (iii) the long unsaturated side chain with 15 carbon atoms endows CD with excellent flexibility and reactivity. The structure is shown in Fig. 1. CD and its derivatives are used as additives in biocomposites,^29^ synthetic plastics,^30,31^ and rubbers^32^ owing to their sustainability, low cost, biodegradability, and significant antioxidant characteristics. Mohapatra and Nando^32^ developed CD grafted natural rubber with a higher molecular weight, a higher cure rate and better physical-mechanical properties than unmodified natural rubber. Chen *et al.*^33^ reported that CD-based derivatives are more efficient than dioctyl phthalate in plasticizing PVC.

CD-derivatives, such as di-functional glycidyl ether epoxy cardanol (ECD) or methoxylated hydroxyethyl cardanol (MeCD), have been used as plasticizers for PLA by Hassouna and coworkers.^34,35^ However, there are no detailed studies of CD as the PLA modifier. CD is a commercial product and meets the “double green” criteria. Importantly, the existence of –OH groups in both CD and PLA implies some compatibility between PLA and CD. Thus, we employ CD as a PLA plasticizer and develop environmentally sustainable PLA/CD blends. In this work, the miscibility, thermal and crystallization behaviors, mechanical properties, processability and cytocompatibility of PLA/CD blends are analyzed and investigated in detail.

## Experimental section

### Materials

The polylactide (PLA, Grade 2003D) was provided by Nature Works and dried for 24 h in a vacuum oven at 60 °C before use. Cardanol (CD, purity of 98%) was purchased from Jining Hongming Chemical Reagent Co., Ltd (China). Antioxidant 1010 was purchased from Sinopharm Chemical Reagent Co., Ltd. Mouse preosteoblasts (MC3T3-E1) were kindly donated by Beijing Jishuitan Hospital (China).

### Preparation of PLA/CD blends

PLA, CD and antioxidant 1010 (0.3 wt%) were mixed at 170–180 °C in a torque rheometer for 10 min at a rotation speed of 80 rpm. The weight ratios of CD to PLA were varied from 0 wt% to 30 wt%. For brevity, the blend containing 10 wt% CD was abbreviated to PLA/CD-10. Finally, the samples were compressed into 1 mm thick sheets at 180 °C for 5 min, and were then cold-pressed.

### Characterization

DSC measurement was carried out under N_2_ with a Q200 differential scanning calorimeter (TA Instruments). The specimens were first heated to 190 °C, then cooled to 0 °C, and finally reheated to 190 °C at 10 °C min^−1^. The quenched specimens were directly cooled to −80 °C, and heated to 190 °C at 10 °C min^−1^. The degree of crystallinity of PLA was calculated using the enthalpy of fusion of 100% crystalline PLA, equal to 93 J g^−1^.^36^ Gold-coated sample surfaces were characterized by a SU8010 scanning electron microscope (Hitachi Co., Ltd) at 5 kV.

Dynamic mechanical thermal analysis (DMTA) was performed on a dynamic mechanical analyzer (PE Instruments) in tensile mode at 1 Hz and at a heating rate of 3 °C min^−1^ from −100 °C to 150 °C. Wide-angle X-ray diffraction (WAXD) was performed on a Rigaku RINT diffractometer with Cu Kα radiation (40 kV, 200 mA) in the 2*θ* range of 5° to 50° at a scan rate of 5° min^−1^. Thermogravimetric analysis (TGA, Q50, TA Instruments) was used to investigate the thermal decomposition behavior. The TGA measurements were carried out from 40 to 600 °C at a heating rate of 20 °C min^−1^ under N_2_.

The tensile properties of the PLA/CD blends were tested by using a RGL-3UA tensile testing machine according to ASTM D638 at a crosshead speed of 10 mm min^−1^. The notched Izod impact tests were performed on a Gotech impact machine according to ASTM D256. Rheological experiments were measured on an ARES-G2 rotational rheometer (TA Instruments) in frequency sweep mode from 0.1 to 100 Hz at 180 °C with a strain of 1%.

Cell viability was measured using mouse preosteoblasts (MC3T3-E1) and 3-(4,5-di-methylthiazol-2-yl)-2,5-diphenyltetrazolium bromide (MTT) assays. All samples were cut into slices, sterilized by washing with 75% ethanol, and then rinsed twice with PBS solution. Samples were exposed to Co^60^ for 15 min and incubated in Dulbecco's modified Eagle's medium (DMEM) at the ratio of 3 cm^2^ mL^−1^ for 24 h at 37 °C. The extract solutions were then filtered (0.22 μm pore size) to eliminate the possible presence of solid particles in the samples. MC3T3-E1 cells were grown in DMEM supplemented with 10% fetal bovine serum (FBS) at a density of 5.0 × 10^4^ cells per well and incubated in 5% CO_2_ under humidified conditions at 37 °C. After the incubation, the medium was replaced by the prepared extract dilution which was used as the new culture medium, while the initial medium itself was regarded as a negative control. The cells were allowed to proliferate for 3 days, and the number of viable cells was determined by adding 5 mg mL^−1^ MTT in culture medium. After a further incubation of 4 h, the medium was aspirated, the formed blue formazan crystals were dissolved in isopropanol (BDH, Poole, England), and the absorbance at 450 nm was determined. All sample extracts were tested at least three times to obtain consistent results. The relative viability was calculated by:Relative cell viability = (*A*_test_ − *A*_0_)/(*A*_control_ − *A*_0_)where *A*_control_ refers to the absorbance of the control wells containing cells with DMEM, and *A*_0_ refers to the absorbance of the solution containing only DMEM. The morphology of the cells after incubation for 3 days was observed using an inverted phase contrast microscope before the MTT testing.

## Result and discussion

### Miscibility and morphology

**Fig. 2:**
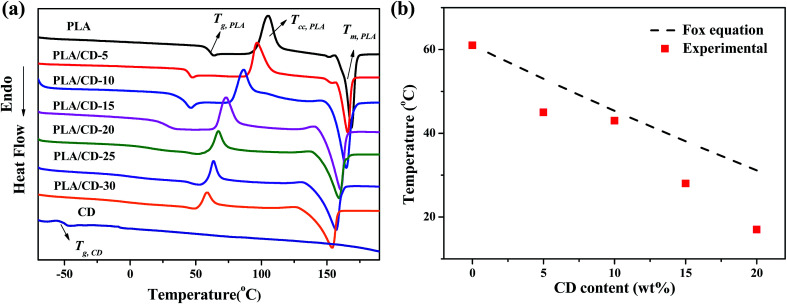
(a) DSC thermograms of PLA, PLA/CD blends and CD; (b) plot of glass transition temperature *vs* CD content.

The miscibility of the polymer blends was characterized and analyzed using the *T*_g_ values of blends of various compositions. We quenched the molten samples by immersion into liquid nitrogen, and the DSC curves obtained from the quenched samples are shown in Fig. 2a. The PLA exhibited a *T*_g_ value of around 61 °C. All blends exhibited only one *T*_g_, suggesting a good miscibility between PLA and CD. The *T*_g_ of the PLA/CD blend dropped significantly with increasing CD content, most likely demonstrating good plasticizing efficiency with PLA. For instance, the *T*_g_ value of PLA decreased by 43 °C and 17 °C for PLA/CD-10 and PLA/CD-20, respectively. As the content of CD was increased to 20 wt%, the *T*_g_ peak became very broad and nearly indistinguishable. Thus, the content of CD in the blends is below 20 wt% in the following discussion.

The *T*_g_ values obtained from DSC are plotted in Fig. 2b according to the Fox equation^37^
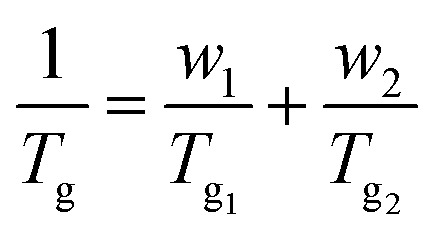
where *w* is the weight fraction and the subscripts 1 and 2 indicate polymer 1 and 2, respectively. The *T*_g_s of the quenched PLA/CD blends show a negative deviation from the empirical Fox equation. It was inferred that small molecule plasticizers move into PLA polymer chains, greatly increase the free volume of the molecular chains, and thus improve the chain mobility of the polymer, resulting in the obvious decrease in *T*_g_.

**Fig. 3:**
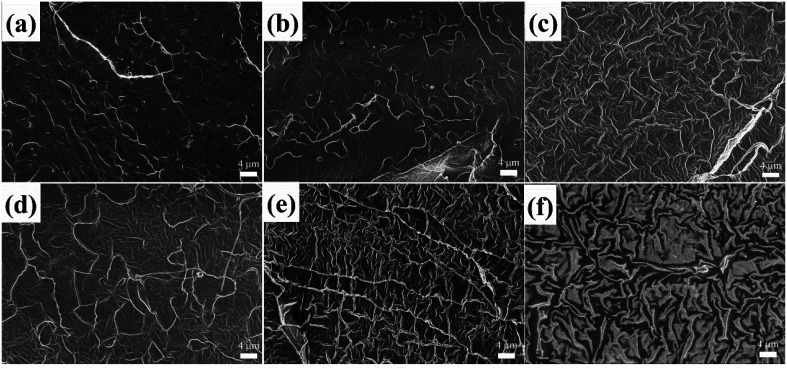
SEM micrographs for cryo-fractured surfaces of PLA/CD blends: (a)PLA/CD-5; (b) PLA/CD-10; (c)PLA/CD-15; (d) PLA/CD-20; (e)PLA/CD-25; (f) PLA/CD-30.

Scanning electron microscopy (SEM) was employed to further evaluate miscibility and phase morphology. Fig. 3 shows the SEM micrographs for cryo-fractured surfaces of the PLA/CD blends. As shown in Fig. 3a and b, PLA/CD-5 and PLA/CD-10 exhibit rough surfaces and few voids can be observed, suggesting that CD is only partially miscible with PLA at lower content. A partially miscible system should exhibit two *T*_g_ peaks in the DSC curves. However, a *T*_g_ peak attributing to CD was not detected, owing to the relatively low CD content in the blends and the limitations of the DSC technique. As we know, the DSC equipment is insensitive to low enthalpy changes at a slow scanning rate. For PLA/CD-15, PLA/CD-20 and PLA/CD-25, no voids can be seen (Fig. 3c–e) and phase separation does not occur, indicating that CD is miscible with PLA. Since small molecule plasticizers migrate toward the surfaces during storage (about 20 days), the surface of PLA/CD-30 is very obscure and phase separation occurs.

### Dynamic mechanical thermal analysis

**Fig. 4:**
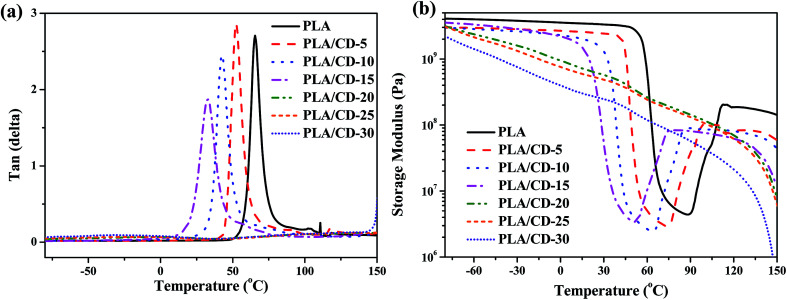
Dynamic viscoelastic curves of PLA and PLA/CD blends: (a) tan δ *versus* temperature; (b) storage modulus *versus* temperature.

Fig. 4 shows the tan delta (tan *δ*) and tensile storage modulus (*E*′) as a function of temperature for the blends. In Fig. 4a, the temperature at the maximum value of tan *δ* corresponds to the *T*_g_. The PLA/CD blends show only one *T*_g_ when the CD content is lower than 15 wt%, and the *T*_g_ values of PLA/CD blends distinctly shift to the lower temperature region with increasing CD content, in line with the aforementioned DSC analysis. The single *T*_g_ for each sample and the reduction in *T*_g_ with increasing CD content both indicate that PLA is miscible with CD. No peak was observed for the PLA/CD blends with CD content higher than 15 wt%, because the blends had already attained high crystallinity.

As shown in Fig. 4b, the *E*′ of PLA drops sharply at around 54–75 °C due to the glass transition, then rises at around 90 °C due to cold crystallization, and subsequently drops again at around 125 °C due to crystal melting. For the PLA/CD blends, the *E*′ curves show two very different trends. When the CD content is lower than 15 wt%, the trend of the *E*′ curves for the PLA/CD blends is similar to that for PLA. The *E*′ at the glassy state gradually decreases with increasing CD content. The temperature at which *E*′ starts to rise shifts to a lower temperature region with increasing CD content, indicating that the introduction of CD enhances the cold crystallization ability of PLA. In contrast, when the CD content is higher than 15 wt%, the *E*′ of the PLA/CD blends decreases with both temperature and CD content. A rise of *E*′ due to the cold crystallization of PLA is not observed, suggesting that the molded sample has already attained almost the ultimate crystallinity before the dynamic viscoelastic test.

### Thermal and crystalline behaviors

**Fig. 5:**
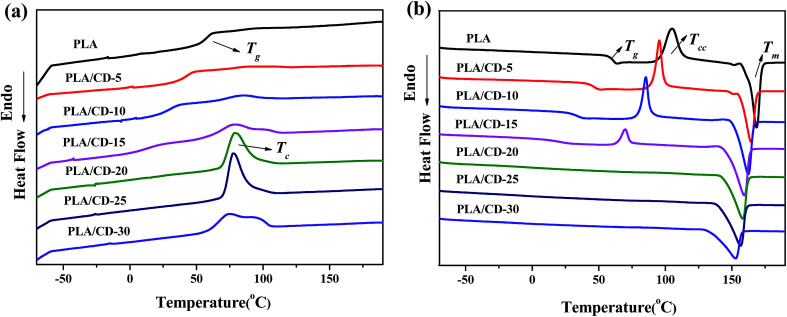
DSC thermograms of PLA and PLA/CD blends: (a) cooling; (b) heating.

The DSC curves of PLA and its blends are exhibited in Fig. 5, and the relevant results obtained from the DSC curves are summarized in Table 1. Upon heating, PLA shows a clear glass transition at 61 °C, a cold-crystallizing peak at 105 °C and a melting peak at 168 °C (Fig. 5b). As shown in Fig. 5b, the PLA/CD blends cold-crystallize at 70–96 °C, and the cold crystallization temperature (*T*_cc_) of PLA decreases as the CD content increases. When the CD content is higher than 15 wt%, the cold-crystallizing peak cannot be observed, but the PLA/CD blends exhibit a sharp and intense crystallization peak during cooling. The higher CD content shifts the crystallizing peak to a lower temperature, and increases the heat of crystallization (Δ*H*_c_) to a higher value. This phenomenon can be ascribed to two main factors: (1) the increased chain mobility which enables PLA to crystallize at a lower temperature upon cooling; (2) the role of the plasticizer as a nucleating agent. Similarly, the melting temperature (*T*_m_) shifts to a lower temperature by 5 to 16 °C and the enthalpy of fusion increases with increasing CD content. In parallel, the degree of crystallinity increases from 1% to 46%, similarly showing that the enhanced chain mobility increases the ability of the PLA chains to crystallize.

**Table 1:** Thermal properties of PLA, PLA/CD blends and CD^a^

CD content (%)	*T*_g_ (°C)	*T*_c_ (°C)	Δ*H*_c_ (J g^−1^)	*T*_cc_ (°C)	Δ*H*_cc_ (J g^−1^)	*T*_m_ (°C)	Δ*H*_m_ (J g^−1^)	Crystallinity (%)
0	61	—	—	105	33.2	169	33.7	0.5
5	45	93	1.0	96	29.9	165	32.6	2.9
10	43	85	6.1	85	25.8	162	37.8	12.9
15	28	78	16.1	70	12.5	159	39.2	28.7
20	15	78	33.1	—	—	158	40.4	43.4
25	5	77	36.5	—	—	157	42.7	45.9
30	−5	74	42.1	—	—	153	43.0	46.2
100	−50	—	—	—	—	—	—	—

a *T*_g_ was obtained from the quenched samples; the values of Δ*H*_c_, Δ*H*_cc_, Δ*H*_m_ were normalized; the crystallinity was calculated by DSC curves.

**Fig. 6:**
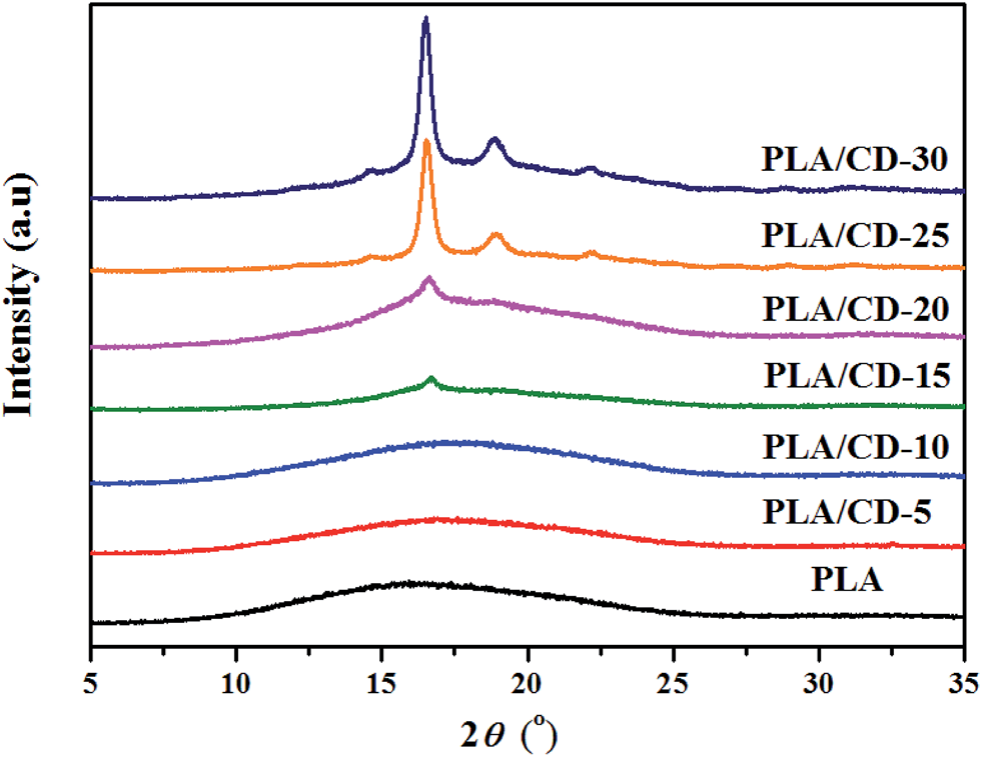
WAXD curves of PLA and PLA/CD blends.

Wide-angle X-ray diffraction (WAXD) spectra were employed to further investigate the crystallization behavior of the PLA/CD blends. As seen in Fig. 6, PLA shows an amorphous structure with no diffraction peaks. PLA/CD blends containing lower CD content, display no diffraction peaks, suggesting that the blends are primarily amorphous. However, diffraction peaks of PLA/CD blends appear and become more intense with the addition of more CD, consistent with the DSC results. When the CD content is higher than 15 wt%, a diffraction peak at 2*θ* ≈ 16.5° appears, corresponding to the (110/200) crystalline plane of α′ or α crystal forms.^38^ In addition, diffraction peaks at around 14.6°, 18.8° and 22.2° are detected, relating to the (010), (203) and (015) planes, respectively.^38^

**Fig. 7:**
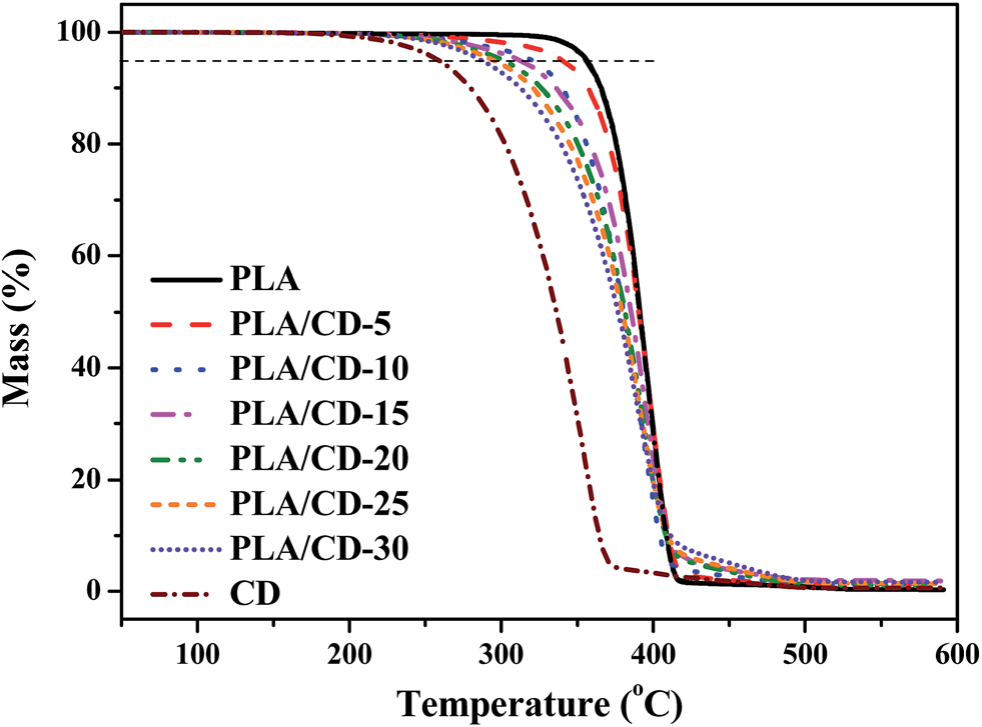
TGA thermograms of PLA, PLA/CD blends and CD.

We also investigated the thermal stability of the PLA/CD blends, and the TGA curves are shown in Fig. 7. The thermal decomposition of neat PLA begins at about 320 °C and reaches a maximum degradation rate at about 398 °C, while thermal decomposition of CD begins at about 220 °C and reaches a maximum degradation rate at about 340 °C. All the blends reveal a single stage decomposition at 290–420 °C, and a maximum degradation rate at 387–398 °C, similar to that of neat PLA. For the PLA/CD blends, the thermal decomposition starts at a lower temperature compared with that of PLA. Therefore, the addition of CD slightly influences the thermal stability of PLA, but it meets the requirements of most applications.

### Mechanical properties

**Fig. 8:**
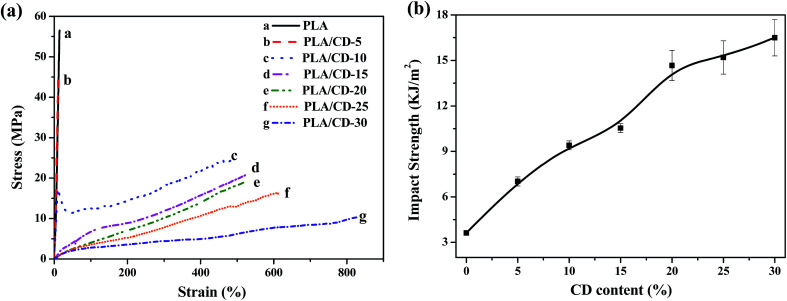
(a) The stress–strain curves of PLA/CD blends; (b) effect of CD content in the blends on impact strength.

The stress–strain behavior of the PLA/CD blends was tested at room temperature, as shown in Fig. 8a. Neat PLA fractures without yielding, exhibiting as a brittle material with a high tensile strength of 52.4 MPa and a low elongation at break of 15%. For PLA/CD-10, a pronounced yield followed by a stable neck is observed; the tensile strength drops to 25.1 MPa and the elongation at break increases to 472%, representing a 31-fold improvement over the value for neat PLA. The increase of CD content to 15 wt% further decreases the tensile strength to about 22.5 MPa, while no yield point is found. Thus, the PLA/CD blends containing more than 15 wt% CD exhibit the stretch behavior of elastomers. Such material flexibility improvement is directly related to the significant increase in chain mobility. The tensile strength and tensile modulus of blends decrease with increasing CD content, which can be reasonably explained by the introduction of small molecules. Thus, the addition of plasticizers causes a decrease in the tensile modulus and tensile strength and an increase in the elongation at break for all the blends. Compared with CD derivatives, the increase in elongation at break of our PLA/CD blend (from 15% to 472%, 31 times) may surpass that found in the previous studies on the toughening of PLA. For example, the elongation at break of a PLA/methoxylated hydroxyethyl cardanol blend increased from 12% to 198%, 16.5 times that of neat PLA.^35^ For a PLA/di-functional glycidyl ether epoxy cardanol blend prepared through *in situ* reactive grafting, the elongation at break increased from 5.7% to 49%.^34^

The impact strength represents the ability of a material to absorb fracture energy during an impact process. The most common and useful measurement of impact strength is the notched impact strength. Therefore, we adopted the notched Izod impact test to characterize the effect of CD on impact toughness. As shown in Fig. 8b, the notched Izod impact strength increases markedly with CD content, and reaches its maximum value at 30 wt% CD. The maximum impact strength is 16.5 kJ m^−2^, a 4.6-fold improvement compared to that of neat PLA.

**Fig. 9:**
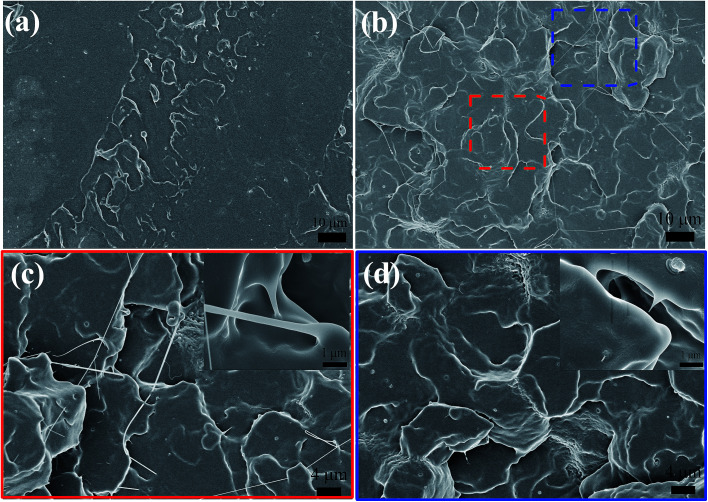
SEM micrographs for impact-fractured surfaces of PLA/CD blends: (a) PLA; (b), (c) and (d) PLA/CD-10.

To identify the toughening effect of CD on PLA/CD blends, the impact-fractured surfaces were studied by SEM. For PLA (Fig. 9a), smooth surfaces with no matrix deformation were found, indicating brittle failure. As shown in Fig. 9b, the fracture surfaces were rough and exhibited some matrix deformation, demonstrating ductile fracture. Under an applied stress, crazes were initiated and propagated outwards to the maximum applied stress. Craze growth was borne by the presence of bridging fibrils (Fig. 9c). For PLA/CD blends, some voids were observed, as shown in Fig. 9d. These voids, forming by cracks, expanded slowly by the breakdown of the surrounding craze fibrils until a void became a crack of a critical size that could propagate catastrophically. PLA/CD blends absorbed more energy than PLA during the process of craze growth, owing to the improved chain mobility.

### Rheological properties

**Fig. 10:**
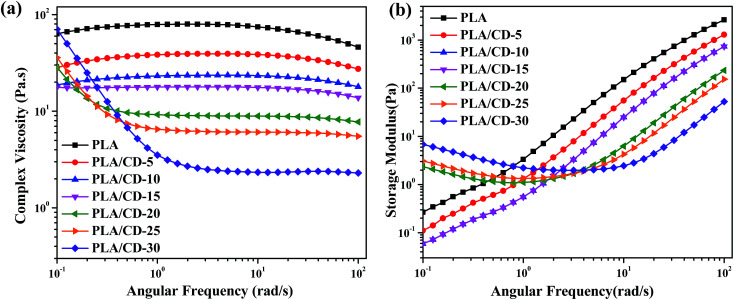
Variation of complex viscosity (a) and storage modulus (b) *versus* angular frequency at 180 °C for PLA and PLA/CD.

To investigate the effect of CD content on the melt rheological properties, frequency sweeps were carried out at 180 °C. Fig. 10a and b present the frequency dependences of complex viscosity (η*) and the shear storage modulus (*G*′) of PLA and PLA/CD blends, respectively. The PLA/CD blends display a lower viscosity and more obvious shear thinning behavior than PLA. Two types of complex viscosity curves can be observed. When the CD content is lower than 15 wt%, an obvious newtonian plateau is observed in the low frequency region and a reduction in complex viscosity occurs in the high frequency region. In contrast, the blends with high CD content (above 15 wt%) exhibit stronger shear thinning behavior with increasing CD content in the low frequency region. The decreased melt viscosity of the blend is ascribed to an increased free volume due to the plasticization by CD.

The shear storage modulus (*G*′) is sensitive to the composition of the PLA/CD blends, and increases with increasing frequency. *G*′ increases with decreasing CD content. The blends with low CD content (below 15 wt%) show lower *G*′ values than PLA across the full frequency range. *G*′ values exhibit an apparent plateau in the low frequency region when the CD content is increased to above 15 wt%. Generally, *G*′ is related to the elasticity of the microstructure, and the enhancement in *G*′ represents the enhanced elastic response of the melt under shear conditions.

### Cytocompatibility

**Fig. 11:**
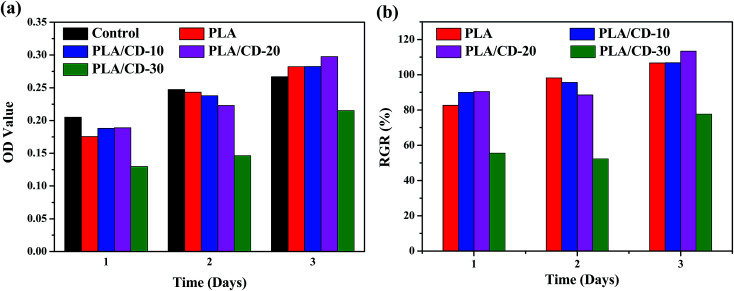
Optical density (OD) value (a) and relative growth rate (RGR/%) (b) of MC3T3-E1 cells cultured on PLA and PLA/CD blends compared with that on control substrate.

**Fig. 12:**
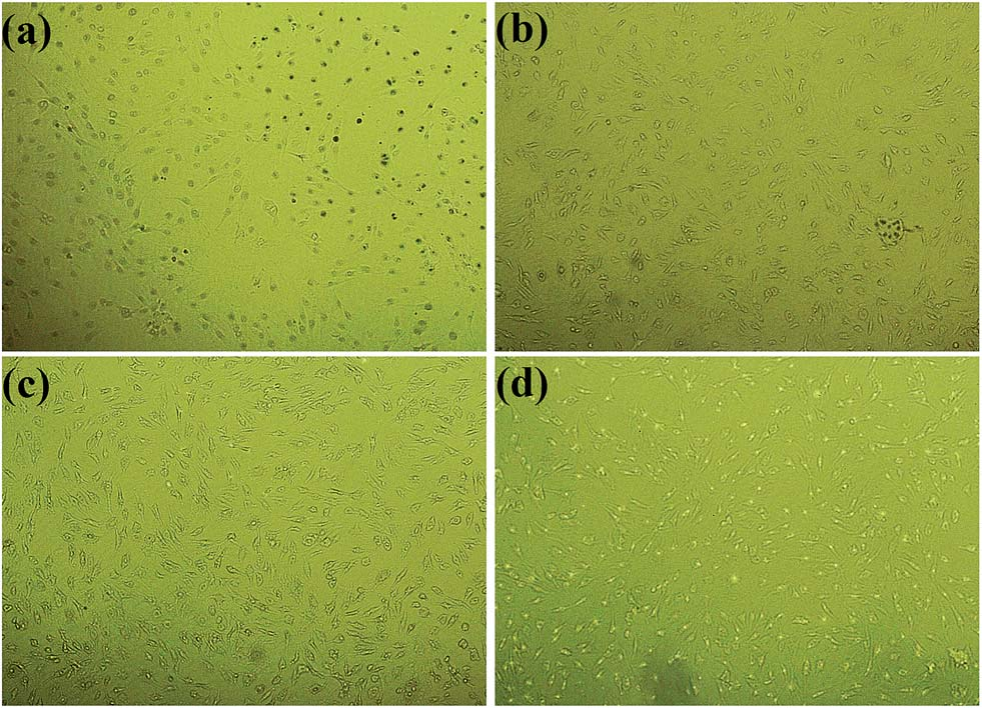
Photomicrographs of MC3T3-E1 cells incubated for 3 days in the negative control (a), and the extract substrates of (b) PLA, (c) PLA/CD-10 (d) PLA/CD-20.

The cytotoxicity of PLA/CD blends was evaluated to determine their suitability for biomedical applications. MC3T3-E1 cells were used in our cytotoxicity assays; the number and morphology of the cells in the extracts were observed. The values of optical density (OD) correspond to the number of live cells in the extract. Based on the cell relative growth rates (RGR) calculated from the OD values, the cytotoxicity of materials can be classified into six grades, shown in Table 2. Grades 0 and 1 mean that the material presents no or very low cytotoxicity to cells, and can be accepted as “qualified” in biomedicine. A material with Grade 2 should be further considered by examining its cell morphology. Other grades are regarded as “unqualified,” indicating that the material presents very high cytotoxicity and cannot be used as a biomaterial. The OD values and RGR for neat PLA and PLA/CD blends are shown in Fig. 11. The OD values of the cells incubated on the substrates increased with time and even outnumbered that from the control after incubation for 3 days. The RGR values of the cells cultured on the blend substrates for 3 days were above 100% and were characterized as grade 0 except for PLA/CD-30. Therefore it is concluded that PLA/CD blends with low CD content show no toxicity to cell viability. As seen in Fig. 12, MC3T3-E1 cells show a normal stellate morphology and exhibit no negative response to PLA/CD blends with low CD content after incubation for 3 days, suggesting no cytotoxicity and the potential biocompatibility of the PLA/CD blends.

**Table 2:** Relationship between cell relative growth rate (RGR) and cytotoxicity grade of a material

RGR (%)	≥100	75–99	50–74	25–49	1–24	0
Cytotoxicity grade	0	1	2	3	4	5

## Conclusions

Renewable blends were prepared by the melt mixing of PLA and CD. DSC and SEM micrographs showed that PLA and CD exhibit good miscibility when the CD content is below 25 wt%. The DSC results showed that blending PLA with CD reduced significantly the *T*_g_ value of the resulting polymer blend, in comparison to that of pure PLA. The crystallization ability of PLA was improved by incorporating CD due to the enhanced chain mobility. The addition of CD slightly reduced the thermal stability of the blends. Considering its good plasticizing efficiency and comprehensive mechanical properties, the PLA/CD blend with 10 wt% CD was identified as the optimal formulation. At this optimal formulation, the elongation at break was significantly enhanced by 31-fold to 472% when compared to pure PLA; meanwhile, the impact toughness was improved by 2.6-fold. The obvious plastic deformation, decreased melt viscosity and shear storage modulus of the blends demonstrated the excellent plasticization effect of CD. *In vitro* cytotoxicity tests showed that these PLA/CD composites are non-toxic towards MC3T3-E1 cells. Collectively, these results demonstrate that cardanol can effectively plasticize PLA; it is a new renewable plasticizer for PLA, holding great potential for biomedical applications.

## Conflicts of interest

There are no conflicts to declare.

## Supplementary Material
